# Minimizing Nonessential Follow-up for Hip Fracture Patients

**DOI:** 10.5435/JAAOSGlobal-D-21-00031

**Published:** 2021-06-02

**Authors:** Michael S. Reich, Julie A. Switzer, Andrew Sibley, Lisa K. Schroder, Sandy Vang, Mai P. Nguyen

**Affiliations:** From the Regions Hospital, Saint Paul, MN (Dr. Reich, Mr. Sibley, Ms. Schroder, Ms. Vang, Dr. Nguyen); the Methodist Hospital, Saint Louis Park, MN (Dr. Switzer); and the University of Minnesota, Minneapolis, MN (Dr. Switzer, Mr. Sibley, Ms. Schroder, Ms. Vang, Dr. Nguyen).

## Abstract

Hip fractures pose a significant burden to patients and care providers. The optimal protocol for postoperative care across all surgically treated hip fracture patients is unknown. The purpose of this study was to investigate the effect that routine follow-up had on changing the clinical course.

**Methods:**

This was a retrospective review of all low-energy hip fractures (ie, femoral neck fractures, pertrochanteric hip fractures, and subtrochanteric fractures) treated surgically from January 2018 through December 2019. Charts were reviewed for demographic information; the procedure performed; the number of postoperative follow-up visits each patient had with the orthopaedic surgery team; the number of sets of postoperative radiographic images obtained; and postoperative complications.

**Results:**

Eight hundred eleven patients with 835 hip fractures were included in the study. The overall number of patient visits was 1,788, and the number of radiograph sets was 1,537. The median number of follow-up visits was two visits/fracture (interquartile range: 1 to 3 visits, maximum = 9 visits), with the median follow-up length of 54 days (interquartile range: 33 to 97 days) with the treating orthopaedic surgeons. Sixty-two (7.6%) patients had 81 (4.5%) postoperative visits and 26 (1.7%) sets of images that led to treatment changes. Among them, 48 (77.4%) patients had concerns that were initiated by the patients and/or care provider. Fourteen standard patient visits led to treatment changes that were not initiated as concerns by the patient and/or care provider.

**Discussion:**

Most clinic visits and radiographs did not lead to a change in the care plan. We recommend that emphasis be placed on comprehensive orthogeriatric care of these patients, and we believe that these data provide the impetus to work toward improving the care pathways for elderly patients with hip fractures.

Hip fractures pose a significant burden to patients and care providers.^1^ Multidisciplinary perioperative treatment plans have been shown to facilitate safe and expeditious surgery, reduce complications, and promote rehabilitation. In addition, routine orthopaedic follow-up is an essential component of care to ensure optimal outcomes. Yet, it is frequently challenging for elderly patients, who may reside at care facilities or at home with family care providers, to attend postoperative clinic visits. In addition, prior work has previously questioned the value of serial postoperative examinations and imaging in fracture care.^2,3,4,5,6^

It is well established that hip fractures commonly occur in elderly patients and are associated with significant morbidity and mortality.^7-9^ Furthermore, studies have demonstrated the benefit that streamlined perioperative care has on promoting positive outcomes.^10-12^ However, a standardized protocol for postoperative follow-up across all patients with surgically treated hip fracture is unknown.

Unlike other lower extremity fractures such as and tibial plateau and pilon fractures, where surgeons may restrict weight bearing and advance it postoperatively based on clinical and radiographic evidence of healing, patients with hip fracture are usually encouraged to weight bear as tolerated immediately after surgery.^13^ Given the need for benevolent care of elderly patients who are frequently frail, optimizing the value of patient encounters postoperatively is essential. Although a multidisciplinary approach with bone health counseling is critical for hip fracture patients, the role of standard postoperative fracture follow-up with orthopaedic surgeons is unclear.

The purpose of this study was to investigate the effect that routine follow-up had on changing the clinical course during recovery for elderly patients with surgically treated hip fractures. We aimed to focus strictly on the effect that postoperative visits had on treatment plans related directly to the patient's hip injury. We hypothesized that most scheduled postoperative visits would not lead to a change in surgeons' treatment algorithms.

## Methods

This study was approved by our institutional review board. Our institutional database was queried for all low-energy hip fractures (ie, femoral neck fractures, pertrochanteric hip fractures, and subtrochanteric fractures) treated surgically from January 2018 through December 2019. The query resulted in 965 patients with 989 hip fractures. Patients with periprosthetic fractures (N = 41 patients), nonsurgically treated fractures (N = 73 patients), patients with less than 1 months' follow-up (N = 39 patients), and one patient with simultaneous bilateral hip surgery were excluded. All other patients were included (N = 811 patients, 835 fractures).

Charts were reviewed for demographic information; the procedure performed; the number of postoperative follow-up visits each patient had with their orthopaedic surgeon; the number of visits with a geriatric orthopaedic nurse practitioner at the patients' residence or care facility; the number of sets of postoperative radiographic images obtained; and postoperative complications. A routine visit was one during which no changes were made to the standard postoperative care of the patient's hip fracture. Essential visits were those during which there was a clinical decision that altered the standard postoperative course, including, but not limited to, a change in weight-bearing status, local wound care or antibiotic prescription, nonroutine laboratory or imaging study order (ie, deep vein thrombosis [DVT] ultrasonography), second opinion referral or specialty consultation, referral to the emergency department or readmission, or determination of an indication for revision surgery. At our institution, counseling regarding metabolic bone disease, management of osteoporosis, and nutritional status is done by trained orthogeriatric physician extenders in one-on-one visits and is vital to caring for these patients, but was not considered an essential surgeon visit in this context.^14^ Statistical analysis was performed with Microsoft Excel.

## Results

Eight hundred eleven patients with 835 hip fractures were included in the study. Of these patients, 562 (69.3%) were female, and the mean age was 83 ± 8.9 years. Complete demographic information is further outlined in Table 1. Most fractures were treated with cephalomedullary nails (N = 343, 41.1%). A total of 308 fractures were treated with arthroplasty, with 62 total hip arthroplasties and 246 hemiarthroplasties. Sixty fractures were treated with closed reduction and percutaneous pinning, 123 fractures with sliding hip screws, and one fracture with a resection arthroplasty (Table 2).

**Table 1:** Demographic Information

No. of fractures	835
No. of patients	811 (24 patients with bilateral fractures in separate admissions)
Male	249 (30.7%)
Age	83 ± 8.9 years old
Body mass index	25.1 ± 5.3

**Table 2:** Surgical Treatment Distribution

No. of primary surgeries	835
Cephalomedullary nail	343 (41.1%)
Hemiarthroplasty	246 (29.5%)
Total hip arthroplasty	62 (7.4%)
Dynamic hip screw	123 (14.7%)
Closed reduction screw fixation	60 (7.2%)
Resection arthroplasty	1 (0.1%)

The overall number of patient visits was 1,788, and the number of radiograph sets was 1,537 (Table 3). The median number of follow-up visits was two visits/fracture (interquartile range: 1 to 3 visits, maximum = 9 visits), with the median follow-up length of 54 days (interquartile range: 33 to 97 days) with the treating orthopaedic surgeons.

**Table 3:** Patterns of Postoperative Visits and Radiograph

No. of visits	1,788
No. of visits that led to a change in treatment plan	81 (4.5%)
No. of radiograph sets	1,537
No. of radiograph sets that led to a change in treatment plan	26 (1.7%)

Sixty-two patients (7.6%), with 62 fractures (7.4%), had postoperative visits that led to treatment changes. Eighty-one clinic visits (4.5%) and 26 sets of images (1.7%) led to a change in the treatment plan. The median number of essential visits was 0 visits/fracture. The most common reasons for visits were for implant-related complications (N = 25, 30.9%) and wound complications (N = 19, 23.5%). These details are further outlined in Table 4. Twenty-seven (3.2%) patients underwent revision surgeries.

**Table 4:** Reasons for Essential Visits

Leg-length discrepancy	6 (7.4%)
Wound-related complications	19 (23.5%)
Implant-related complications	25 (30.9%)
New diagnosis/report	5 (6.2%)
Weight-bearing restrictions	1 (1.2%)
Corticosteroid injections	10 (12.3%)
Venous duplex	3 (3.7%)
Pain	4 (4.9%)
Nonunion	4 (4.9%)
Instability	4 (4.9%)
Total no. of essential visits	81

Among the 62 patients (62 fractures) who had at least one essential visit, 48 (77.4%) had concerns, and subsequently clinic visits, that were initiated by the patients and/or care provider. These concerns included an increase in pain, wound issues, new trauma, or leg-length discrepancy. In an additional 14 patients, scheduled, routine visits became essential visits: seven patients were diagnosed with implant-related complications or nonunions, three patients were diagnosed with wound complications, and one patient was diagnosed with a DVT. One additional patient had weight bearing advanced after initially being treated with restricted weight bearing, and two patients were assessed for DVT and were negative.

Seven hundred forty-nine patients (773 fractures) presented for routine follow-up without changes made to their care plan. In 15 of these cases, either the patient or their nonorthopaedic care provider expressed concern leading to the adverse outcome diagnosis outside of their clinic visits, including 13 visits to the emergency department due to acute pain, one wound complication reported by a patient's care facility team, which was treated remotely by the surgeon, and one implant failure diagnosed after inability to participate with physical therapy. Adverse outcomes among these cases included failure of fixation (N = 8 patients), wound complications (N = 5 patients), and hip instability (N = 2 patients). Eleven subsequent revision surgeries and 12 readmissions related to patients' hip fractures occurred.

## Discussion

The care of elderly patients with hip fractures is complex and involved. These patients face unique challenges that warrant special consideration. Here, we found that the postoperative care plan developed for each patient at the time of surgery went uninterrupted in the overwhelming majority of patients.

Prior work has raised doubt as to the necessity of routine postoperative visits after hip fracture surgery. In a series of Finnish hip fracture patients focusing on the first postoperative visit, Kuorikoski and Soderlund^4^ found that the first visit led to a change in treatment course for one patient out of 423 consecutive patients. In another Finnish study, 7.3% of patients with hip fractures treated with cephalomedullary nails had their postoperative care changed, with most adverse outcomes being diagnosed at unplanned visits.^3^ These findings are consistent with our findings that the treatment of 77 patients (9.4%) deviated from the standard postoperative course, with the complication diagnosed in clinic for 7.4% of fractures. Importantly, for approximately 80% of patients with a surgical complication, either the patient or a care provider reported a concerning symptom leading to the diagnosis. These findings support the notion that the patients with concerns should be followed closely by their surgeons, but for the remaining patients, routine care is unlikely to lead to a change in one's treatment plan.

Only 14 of 811 patients (1.7%) had treatment changes that were not in response to a concern presented by the patient or care provider and were made during a standard follow-up visit. Half of these patients had complications related to their implant or lack of healing, which should have led to persistent or residual pain, but this could also be a symptom consistent with a well-healing fracture and uncomplicated recovery. This scenario presents a challenge in counseling patients/caregivers on postoperative expectations, but also highlights the importance of closer follow-up when patient feedback may not be ideal.

In light of these findings, emphasis could be placed on these patients' global recovery including metabolic bone disease treatment and fracture prevention strategies. Such treatment is consistent with recommendations provided by the American Orthopaedic Association and American Academy of Orthopaedic Surgeons.^15,16^ Initial osteoporosis assessment and intervention can be made while a patient is hospitalized with a hip fracture and can be continued as an outpatient by an orthogeriatric care team or fracture liaison service.^17-19^

It should be noted that in our study cohort, nearly 30% of our hip fracture patients were cared for by a dedicated orthopaedic geriatric outreach advanced practice provider, a member of the orthopaedic surgery team.^20^ These postoperative patient visits, although primarily devoted to postoperative fracture management, also included counseling on the importance of nutrition and lifestyle choices, pharmacologic treatments for osteoporosis, bone mineral density assessment, and rehabilitation and fall prevention. Families were almost always involved in this care provision and counseling.

Suggesting that no orthopaedic follow-up be provided after surgical hip fracture care is not the intent of the analysis of these data. Rather, consideration of more innovative follow-up care that does not involve a standard clinic visit with the operating surgeon, but that addresses the needs and preferences of these patients and their families, is crucial to providing best, comprehensive post–hip fracture care. The cost and time required for using necessary transportation options and the possible development of anxiety and confusion related to transitioning from the nursing care environment to the clinic should be considered when determining the potential benefit of routine postoperative clinic visits. In addition, for patients who are recovering well, longer term follow-up with the surgeon is rarely indicated; approximately 25% of clinic visits in this study occurred outside of the postoperative global period, and the charges associated with an established patient visit including imaging (current procedural terminology codes 99213 and 73502) at our institution are approximately $375. More creative ways to optimize the treatment and postoperative care of hip fracture patients are warranted.

Interpretation of the data presented should be done in the context of the study's limitations. As this is a retrospective case series, the study lacks a comparative control group, and the data are susceptible to selection bias. Only data contained in our electronic medical record system were available for our review, and patient encounters and complications occurring outside of our hospital system would not be captured. However, of 850 patients eligible for inclusion, only 4.6% were excluded due to lack of follow-up, and thus, we were able to follow most of our patients. We also did not include any adverse outcomes diagnosed by patients' primary care providers, and we do acknowledge that they frequently diagnose and treat minor adverse outcomes without consulting the surgical teams. However, this fact would support our conclusion that much of the necessary care of these patients occurs outside of the surgeon-patient clinic encounter. The data presented do not include patient-reported outcomes. We attempted to assess patient-reported outcomes for this population; however, only 17% of patients completed their surveys, thereby precluding any meaningful conclusions.

In conclusion, although the importance of perioperative care of elderly hip fracture patients cannot be understated, we question the value of repeated postoperative clinic visits and radiographs. Certainly, routine surveillance and monitoring of surgical wounds and fracture healing has its value, but we recommend that consideration be made for minimization of the occurrence of this in a standard orthopaedics clinic unless the patient or care provider informs the team of a concern or impending complication. We recommend optimizing the patient experience by incorporating bone health and fracture prevention care into their visits. When possible, consider on-site postoperative follow-up or virtual visits to minimize the burden of transport to a clinic visit. Most clinic visits and radiographs did not lead to a change in the care plan ascribed at the time of surgery, and as such, this observation presents an opportunity to improve the care pathways for elderly patients with hip fractures.
